# Sensitive Dual Color *In Vivo* Bioluminescence Imaging Using a New Red Codon Optimized Firefly Luciferase and a Green Click Beetle Luciferase

**DOI:** 10.1371/journal.pone.0019277

**Published:** 2011-04-22

**Authors:** Laura Mezzanotte, Ivo Que, Eric Kaijzel, Bruce Branchini, Aldo Roda, Clemens Löwik

**Affiliations:** 1 Department of Pharmaceutical Sciences, University of Bologna, Bologna, Italy; 2 Department of Endocrinology and Metabolic Diseases, Leiden University Medical Center, Leiden, The Netherlands; 3 Department of Chemistry, Connecticut College, New London, Connecticut, United States of America; Cinvestav, Mexico

## Abstract

**Background:**

Despite a plethora of bioluminescent reporter genes being cloned and used for cell assays and molecular imaging purposes, the simultaneous monitoring of multiple events in small animals is still challenging. This is partly attributable to the lack of optimization of cell reporter gene expression as well as too much spectral overlap of the color-coupled reporter genes. A new red emitting codon-optimized luciferase reporter gene mutant of *Photinus pyralis*, Ppy RE8, has been developed and used in combination with the green click beetle luciferase, CBG99.

**Principal Findings:**

Human embryonic kidney cells (HEK293) were transfected with vectors that expressed red Ppy RE8 and green CBG99 luciferases. Populations of red and green emitting cells were mixed in different ratios. After addition of the shared single substrate, D-luciferin, bioluminescent (BL) signals were imaged with an ultrasensitive cooled CCD camera using a series of band pass filters (20 nm). Spectral unmixing algorithms were applied to the images where good separation of signals was observed. Furthermore, HEK293 cells that expressed the two luciferases were injected at different depth in the animals. Spectrally-separate images and quantification of the dual BL signals in a mixed population of cells was achieved when cells were either injected subcutaneously or directly into the prostate.

**Significance:**

We report here the re-engineering of different luciferase genes for *in vitro* and *in vivo* dual color imaging applications to address the technical issues of using dual luciferases for imaging. In respect to previously used dual assays, our study demonstrated enhanced sensitivity combined with spatially separate BL spectral emissions using a suitable spectral unmixing algorithm. This new D-luciferin-dependent reporter gene couplet opens up the possibility in the future for more accurate quantitative gene expression studies in *vivo* by simultaneously monitoring two events in real time.

## Introduction

During the last decade, bioluminescent (BL) imaging has become an indispensable tool for visualizing molecular events at a cellular level both *in vivo* and *in vitro* leading to new advances and discoveries in life sciences [1].

There are many available BL luciferase/luciferin reporter gene systems for *in vivo* imaging:the first reportedly used, and most popular, are the luciferases that require D-luciferin and are ATP dependent (i.e. firefly luciferase, click beetle luciferase) [2].Other luciferases followed such as Renilla luciferase and Gaussia luciferase which require coelenterazine as a substrate and are ATP independent [2], [3]. In addition, the use of the blue emitting (490 nm) bacterial luciferases from *Photorhabdus. luminescens* has been reported [4]. Such luciferases do not require the infusion or administration of the BL substrate and are scarcely expressed in mammalian cells. The codon-optimized version of this luciferase has been recently proposed for *in vivo* imaging but it is less robust than firefly luciferase [5].

Renilla and Gaussia luciferases emit blue light which in part compromise their *in vivo* performance due to extensive light absorption by the small animal body. Blue light is strongly absorbed by tissue components particularly in highly vascularised tissues where haemoglobin is present [6].

In the case of Renilla, new red-shifted and more stable mutants with an emission peak at 535 or 547 nm have been produced by site directed mutagenesis [7], but dual color imaging still remains difficult to perform in part due to the relative low quantum efficiency of CCD cameras below 500 nm (30%), where the native enzyme shows the peak of emission.

Until now there are no red-shifted Gaussia luciferase mutants available but only brighter ones or with a prolonged half-life [3], [8], [9]. Red-emitting mutants from the railroad worm (*Phrixothrix hirtus*) , possessing higher activity and better stability. have recently been proposed for BL imaging but these have not yet been fully investigated for *in vivo* applications [10].

Click beetle and firefly luciferases, variants with different emission wavelengths have been developed but they do not possessoptimal characteristics for *in vivo* imaging [11], [12]. In particular, red/green couplets of reporter proteins for *in vivo* applications must possess intense BL emission with narrow emission spectra resulting in a reasonable separation and with good thermostability at 37°C [12], [13], [14].

Codon-optimization of the reporter gene is a fundamental prerequisite for improving the BL signal in mammalian cells thus facilitating their detection *in vivo* [15].

Recently it has been reported that dual color BL imaging could be applied *in vitro* using appropriate filters for the separation of BL signals and mathematical corrections for their deconvolution [16], [17]. Furthermore, *in vivo* applications using multicolor analysis can be achieved using different substrates or fluorescent proteins [18].

The fate of two different cell populations, was simultaneously monitored in vivo when the novel red codon-optimized luciferase reporter gene mutant of *Photinus pyralis*, Ppy RE8, was combined with the green click beetle luciferase, CBG99. Ppy RE8 is characterized by a peak emission at 618 nm and has an excellent thermostability (half-life of 4,5 h at 37°C) [13]. CBG99 is a pH insensitive luciferase with an emission maximum at 537 nm which showed better performance for *in vivo* applications than the widely used *P. pyralis* wild type luciferase (PpyWT) [19].

Here, we demonstrate the applicability of the two luciferases *in vitro* and *in vivo* by generating lentiviral vectors for the expression of the genes under the control of the CMV promoter. Multicolor HEK293 cell-based assays were developed to evaluate the suitability of simultaneous measurements of the red and green emitting luciferases by spectral unmixing. Both luciferases maintained the same spectrum of emission in cells at 37°C. We also show the applicability of the dual luciferases *in vivo*, after intraperitoneal injection of D-luciferin, when HEK293 cells were inoculated either subcutaneously or injected directly into the prostate in mice and then imaged. A good separation of the individual signals could be obtained using spectral unmixing algorithms for their deconvolution. Ppy RE8 was demonstrated to be an excellent tool for *in vivo* BL imaging and, in particular, when used in combination with a green luciferase to monitor dual events at the molecular level. The use of a single D-luciferin substrate for the same couple of reporter gene allows time and cost saving in contrast to dual-color luciferase imaging using Firefly and Renilla luciferases in which the addition of a second luminescent substrate, coelenterazine, is warranted.

## Results

### Emission spectral unmixing of bioluminescence in cell lysates

**Figure 1:**
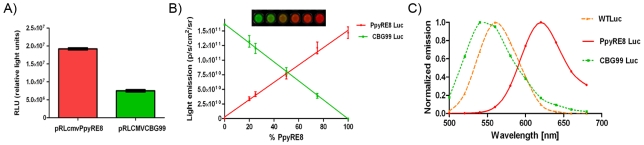
Validation of Ppy RE8 and CBG99 as a bioluminescent couple for multicolor imaging. (A) Level of expression of lentiviral constructs in HEK293 cells. (B) Spectral unmixing of cell lysates mixed in different proportions. Cells were lysed 24 h after transfection with lentiviral constructs. (C) Emission spectra of luciferases calculated with Living Image software in cell lysates at 25°C with the Ppy RE8 peak around 620 nm, CBG99 around 540 nm and WT Luc around 560 nm.

**Figure 2:**
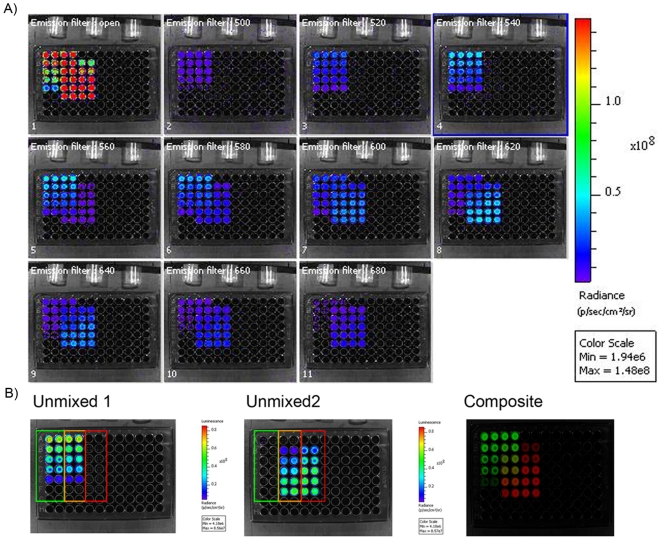
Representative image of emission spectral unmixing of bioluminescence in cell lysates. (A) Multispectral acquisition of red and green emitting cell lysates. In the left part (row 1 and 2) of the plate dilutions of green emitting lysates were dispensed in duplicate while in the right part (row 5 and 6) dilutions of the red ones. In the middle (row 3 and 4) lysates were mixed in different proportions. The plate was scanned with an open filter and at different wavelengths ranging from 500 nm to 680 nm with a 20 nm interval.(B) Resulting unmixed images used for calculation and composite of the two different luciferases in false colors.

The ability of the two red and green selected luciferase signals to be detected and quantified in a single run, using a single substrate, was evaluated. The red codon-optimized luciferase reporter gene, Ppy RE8, and the green emitting click beetle, CBG99, were expressed transiently under the control of the same promoter in HEK293 cells and lysed after 24 hours. For the same number of cells the light output of red emitting lysate was 2.5 higher than the one of green emitting cells (Fig. 1A). In our constructs and assay conditions, Ppy RE8, produced more light that CBG99. Both luciferases produced more signal than CBred when cloned in the same plasmid backbone and expressed in HEK293 cells, as previously reported [11], [13]. Moreover, when cell lysates of the red or green expressing cells were plated in different ratios, calculations of the percentages of red and green light in a mixture were possible by applying the spectral unmixing algorithm to the acquired images (images acquired using a series of 20 nm band pass filters) as shown in Figure 1B. In this set of experiments there were no significant differences between plated and calculated percentage of cell lysates which demonstrated the validity of the method. In addition, the algorithm allowed for the calculation of the emission spectra for both luciferase variants plus the Ppy WT, which were similar as those obtained when analysed separately (Fig. 1C). A representative image of a spectral unmixing of cell lysates is shown in Figure 2. Lysates from red and green expressing cells were serially diluted in duplicate and mixed in different proportion. In Figure 2A images were taken using a series of band pass filters (20 nm) and as a control in the absence of any filters. Figure 2B shows the unmixed images corresponding to the red and green signals. Data were calculated from the unmixed images that corresponded to the red and green signals.

### Live cell dual color imaging

**Figure 3:**
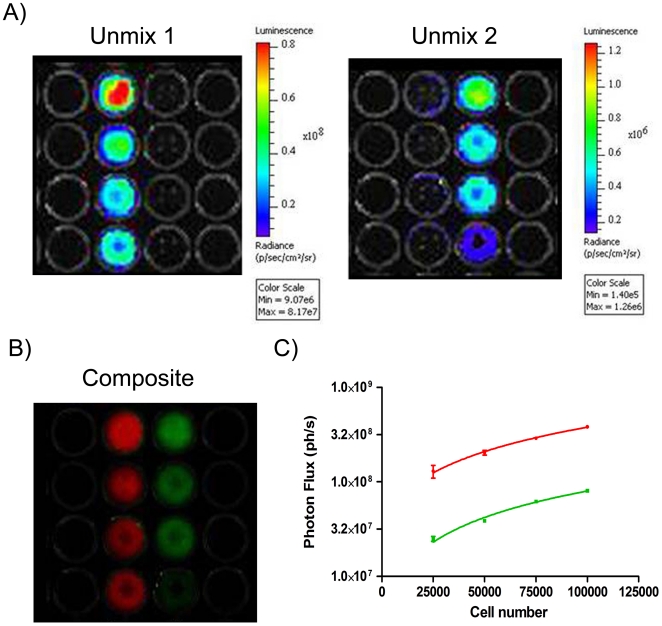
Live cell imaging. (A) Representative spectral unmixing of signals emitted from stable red and green HepG2 cells;10^5^, 7.5×10^4^, 5×10^4^ and 2.5×10^4^ cells were plated for each HepG2 cell line. (B) Composite images generated after unmixing signals. (C) Graph representing the correlation between luminescent signals and different amounts of red or green luciferase expressing HepG2 cells.

In this set of experiments, HepG2 cells were stably transduced using lentiviruses that expressed the different luciferases. This was used to evaluate the performance of this pair of luciferases in living cells. Selected clones of cells stably expressing the luciferase variants cannot mirror the expression level of transiently transfected populations, and different promoters vary expression in different cells types. For these reasons a direct comparison between the level of expression of CBG99 and Ppy RE8 luciferase could not be performed but other relevant parameters such as emission spectra and dynamic range of luminescence signals for *in vivo* application could be evaluated. Ppy RE8 expressing cells showed a 5-fold higher signal than the cells expressing CBG99 at 37°C. Figures 3A and 3B report a representative analysis of different amounts of the red and green HepG2 cells at 37°C when an unmixing algorithm was applied to the acquired images. Analysis of the images allowed us to determine that the emission spectra of Ppy RE8 and CBG99 cells did not vary in intact cells at 37°C under physiological pH conditions. A good correlation between number of cells and light emission was obtained (R^2^  = 0.98) when the experiment was carried out in triplicate (Fig. 3C).

### *In vivo* dual color imaging

**Figure 4:**
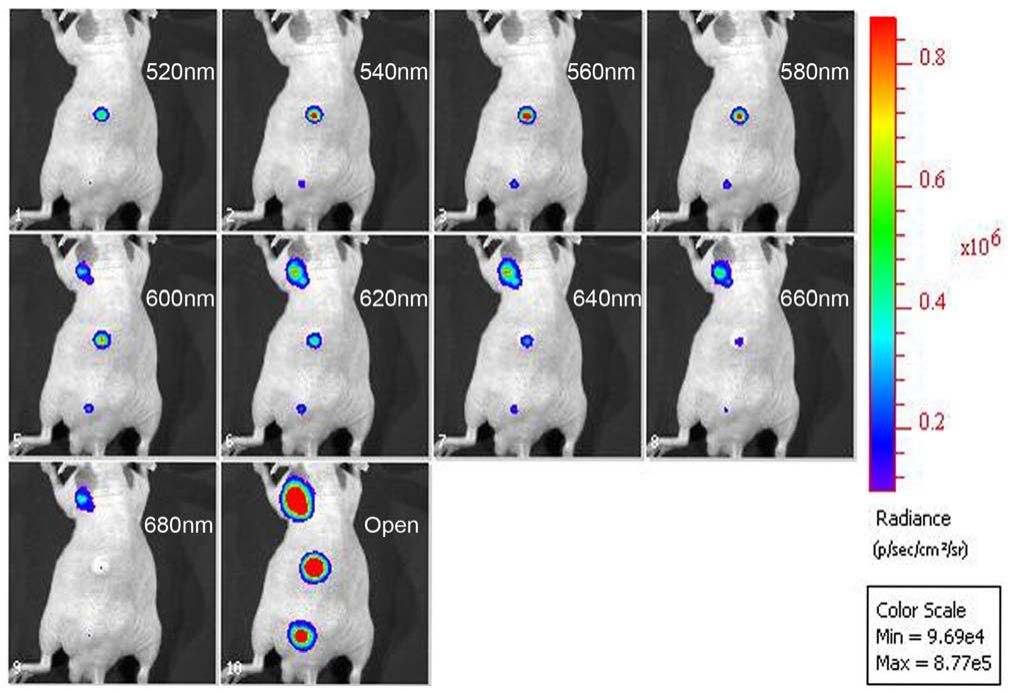
Multispectral acquisition of light from live animal. Cells expressing Ppy RE8 and CBG99 luciferases and a mixture were inoculated in the upper part, middle part and lower part of the back, respectively.

**Figure 5:**
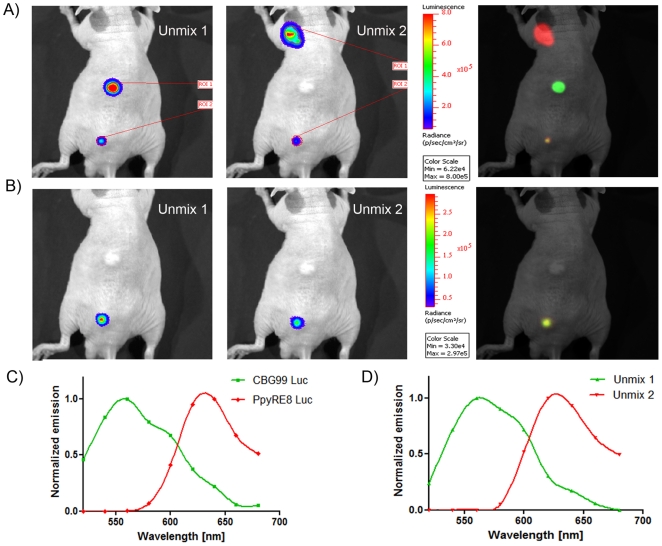
Spectral unmixing of signals after subcutaneous injection of cells. (A) Unmixed and composite images. The injected cells were 10^5^ (upper and middle part) and 2.5×10^4^ in the mixture (lower part). The numbers of cells calculated with Living Image software were 2.0±0.4×10^4^ for CBG99 and 2.4±0.2×10^4^ for Ppy RE8. B) Unmixed and composite image generated from the ROI of the mixture. C) Emission reference spectra of luciferases calculated from the *in vivo* experiments. CBG99 spectrum is represented with a green line while Ppy RE8 spectrum is represented with a red line. A slight red shift was noticed for both luciferases. D) Unmixed spectra calculated for the region of interest of the mixture. The spectra are almost identical to the reference ones.

In order to test this pair of luciferases for *in vivo* applications, cells expressing each luciferase were injected subcutaneously in mice. Five minutes after substrate injection, a series of images with 30sec acquisitions were obtained. The emission spectra of the luciferases calculated from the *in vivo* experiments showed a slight red shift because of absorption and scattering of light generated under the skin (Fig. 4). Intensity of the BL signals allowed the calculation of the red/green cell ratio in a mixed population after applying spectral unmixing algorithm. Average luminescence expressed in photon/sec/sr/cm^2^ was determined for the ROI corresponding to the different areas where cells were inoculated. These values were extracted from the unmixed images generated by Living Image software. Experiments carried out in three mice for both independent set of experiments gave reproducible results. Cells (10^5^) expressing only the green or red luciferase were subcutaneously injected in the upper and middle part of the back. The mixed population of red and green emitting cells was composed by 2.5×10^4^ cells of each population.,The calculated numbers of cells in the mixture were 2.0±0.4×10^4^ for CBG99 and 2.4±0.2×10^4^ for Ppy RE8 (Fig. 5A). These data were confirmed when the algorithm was applied only to the ROI corresponding to the mixed population. Furthermore, the data generated were concordant to that generated in whole body scans of the mouse which confirmed the good unmixing spatially of red and green signals. Moreover the normalized spectra obtained from this region (Fig. 5D) under the skin were almost identical to the reference spectra calculated from cells emitting only one luciferase (Fig. 5C).

**Figure 6:**
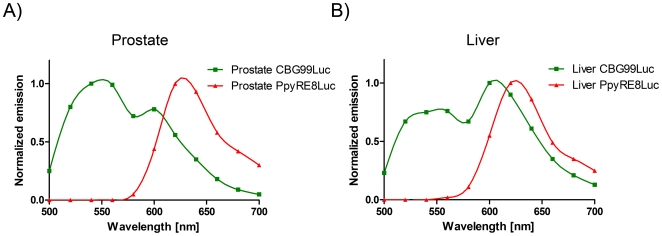
Reference spectra calculated after injection of red or green emitting cells in the prostate (A) and in the liver (B). CBG99 spectrum is represented with a green line while Ppy RE8 spectrum is represented with red line.

**Figure 7:**
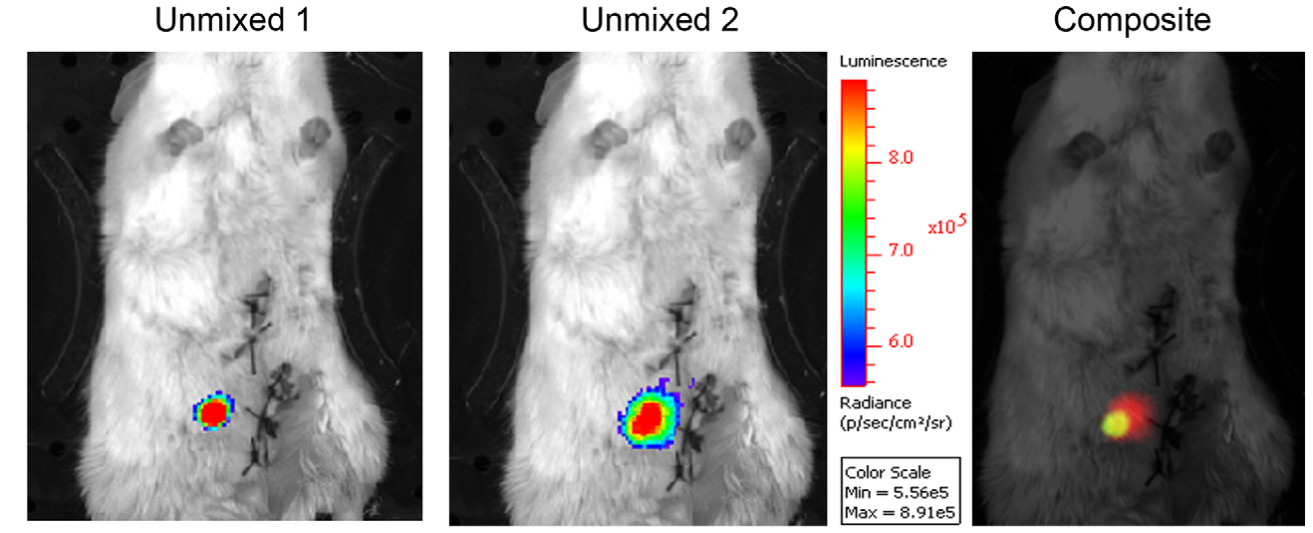
Representative unmixing images generated after injecting a mixture of red and green cells in the prostate.

**Figure 8:**
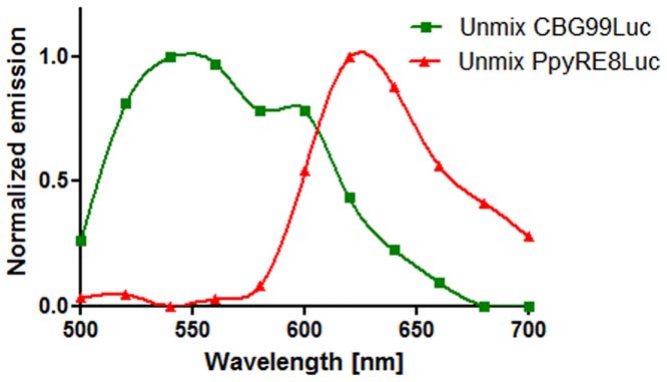
Unmixed spectra calculated for the region of interest of the mixture. CBG99 spectrum is represented with a green line while Ppy RE8 spectrum is represented with red line.

Further experiments were conducted to investigate the use of the red/green luciferase couplet at different depths and in tissues with different absorption properties whereby red or green emitting HEK293 cells were injected in the liver and in the prostate. The calculated normalized spectra are reported in Figure 6. The spectrum of emission of Ppy RE8 did not vary considerably while the spectrum of the CBG99 changed. In the liver (Fig. 6B), the CBG99 spectrum became bimodal with a peak around 600 nm and a shoulder at a lower wavelength. This is likely due to the presence of large amounts of hemoglobin, the principal absorber of green light, which prevents good spectral unmixing of the signals within this organ. When injected in the dorsolateral prostate, the signal output was lower but the spectrum shape of CBG99 still produced good spectral unmixing (Figure 7). The spectra obtained from the unmixed ROI shown in Figure 8 were comparable to the reference spectra.

## Discussion

The main advantages of using BL in bioanalysis are related to the high signal/noise ratios and quantum efficiencies of the luciferin/luciferase system. This gives rise to sensitive cell based assays and for in vivo molecular imaging [20], [21]. Moreover, the availability of luciferases with different emission wavelengths gives the possibility of performing multicolor and multiplexed assays. Here, we evaluated for the first time a new red-codon optimized luciferase, Ppy RE8, in combination with a green click beetle, CBG99, luciferase that permitted a simultaneous, sensitive and reliable 2D imaging and quantification of different imaging signals* in vivo *using the same D-luciferin substrate. Issues concerning* in vivo *applications, such as cell tracking in deep tissues, are different from that concerning analysis of gene expression in cell based assays. For this reason, we carried out experiments in three different conditions:in cell lysates, in live cells and in whole animals. In order to demonstrate its performance we employed the Ppy RE8 and CBG99 genes for the development of lentiviral expression vectors and used them for transient and stable expression in different cell lines. A major concern was to separate the green emission overlap with the red filter (620 nm) particularly when the two signals have a different intensity. Images were obtained by collecting light using a set of filters (20 nm band pass) from 500 nm to 680 nm and without a filter. This was performed on the IVIS Spectrum (CaliperLS Inc, Hopkinton, MA) and a spectral unmixing algorithm was applied to all the images using the Living Image 4 software (CaliperLS, Inc). This unmixing algorithm enabled the deconvolution of the luciferases as two distinct spectra. The spectral unmixing of the images obtained from cell lysates showed the suitability of the use of these red and green luciferases as a BL pair with a single substrate. Images were collected five minutes after substrate addition when signals of both emitting enzymes are stable as indicated by previous studies [11], [13]. An analysis was performed on stable transfected HepG2 cells to mimic the conditions for* in vivo *imaging. In this case, temperature was set to 37°C and the substrate consisted of 1 mM D-luciferin without cofactors normally present in commercial assay buffers for testing cell lysates. Each image generated by a different band pass filter of 20 nm was obtained by integrating signals for 30 s since ATP present in living cells represents a limiting factor on bioluminescence intensity. In this set of experiments, acquisition of images took 5 minutes and generated accuracy of detection and quantification. Moreover, we envisage the possibility to perform single cell or tissue analysis by using a novel implemented microscope for dual color bioluminescence imaging [22], [23]. To complete the evaluation and to establish that the red/green luciferase couplet is optimal for* in vivo *imaging , studies of the luciferase pairing were performed in living animals. Our results from living mice inoculated with red and green emitting cells demonstrated the possibility for 2D visualization and (semi-)quantification of cells which produced different colors in mixed populations. The light emission of both luciferases underwent a red-shift of 20 nm due to tissue absorption and scattering of light generated under the skin. As previously reported [24], emission spectra of luciferases* in vivo *are affected by tissue depth and concentration due to absorption and scattering of light through tissues. Therefore, our luciferase couplet will be adequate for studying cells implanted subcutaneously or in the mammary fat pad for breast cancer research. Moreover, the use of different organisms for cancer research such as transparent adult zebrafish [25] or transparent frogs [26] that can fit in the bioluminescence imager is possible. The experiments carried out in the dorsolateral prostate and in the liver showed that the use of the red emitting luciferase is preferential for imaging in deeper tissues since the spectrum of emission does not change and the attenuation is lower. Regarding the green emitting luciferase, the spectrum of emission varies due to the presence of absorbers like haemoglobin but it did not hamper a good separation of signals in the dorsolateral prostate. Moreover, future improvement of the analytical performance of spectral unmixing of light signals as in fluorescence applications should lead to a better separation in deeper tissues [27]. Recently, Hida and colleagues applied multicolor luciferases to study protein-protein interaction and proposed* Phrixothrix hirtus *red luciferase (em. Max. 630 nm) as an internal control in combination with fusion proteins constructed of different N or C parts of luciferases for a complementation assay. However, no multispectral image acquisition was performed and no unmixing algorithm was applied to images in order to obtain effective quantification of signals* in vivo* [28].

In conclusion, Ppy RE8 was demonstrated to be an excellent tool for both* in vitro *and* in vivo *bioluminescence imaging and, in particular, when used in combination with a green luciferase to monitor dual events at the molecular level. Ppy RE8 has a good thermostability at 37°C and is highly expressed in mammalian cells. In contrast, the combined use of Firefly and Renilla luciferase requires the use of different substrates that are luciferin and coelenterazine. Biodistribution and enzyme kinetics with the two substrates are very different making ratio-metric measurements more difficult. Therefore, the described new D-luciferin-dependent red/green couplet will allow clearcut (semi-) quantitative gene expression studies* in vivo *and enable simultaneous tracking of different populations of stem cells, T-cell accumulation in tumors and simultaneous analysis of different molecular pathways. An eventual derivation from this study will be to generate new dual color transgenic animal models.

## Materials and Methods

### Ethics statement

Animal experiments were reviewed and approved by the Bioethics Committee of Leiden University, The Netherlands (Animal protocol 08158). Allanimals received humane care in compliance with the ‘Code of Practice Use of Laboratory Animals in Cancer Research” (Inspectie W&V, July 1999).

### Plasmid construction

Self-inactivating lentiviral vectors, pLV.CMV.bc.NEO and pLV.CMV.bc.PURO, were kindly provided by Prof. R. Hoeben. The pLV.CMVPpy RE8.NEO vector was constructed by amplifying the Ppy RE8 gene from pGex Ppy RE8^11^, using the following pair of primers:Ppy RE8ForAscI:taggcgcgccgaggacgccaagaacatca and Ppy RE8RevXhoI:aatctcgagtcagatcttgccgcccttctt, and inserted in the MCS of pLV.CMV.bc.NEO. pLV.CMVCBG99.PURO was created by inserting the CBG99 gene, cut with *Nco*I and *Xba*I from the pCBG99basic vector (purchased from Promega, Madison, WI, USA), into the MCS of pLV.CMV.bc.PURO via blunt ligation.

### Cell lines

HEK293 and HepG2 cells were cultured in DMEM (Sigma, St. Louis, MO, USA) with 10% fetal bovine serum and 2 mM L-glutamine. The cultures were incubated at 37°C with 5% CO_2_. HepG2 cells were transduced by self-inactivating lentiviruses as previously described [29]. Cell clones were selected with 1 mg/ml G418 or 1 µg/ml Puromycin for 14 days.

### Imaging

All images were acquired with an IVIS spectrum (Caliper Life Sciences, Hopkinton, MA, USA) with the stage heated to 37°C during live cell imaging. The plates used were black-walled with clear bottoms. Generally, images were acquired at binning 8×8 pixels, f/stop 1, 12.5 cm field of view for the time and with the filter sets indicated. Experiments carried out with a different setup are indicated. Living Image 4 software was employed for generating spectral unmixed images and calculations of signals.

### Spectral unmixing of emission wavelengths in cell lysates

Confluent HEK293 cells from a T25-flask were trypsinized and 10^5^ cells/well plated in a 6 well plate. The next day, cells were transfected with 1 µg of pLV.CMVPpy RE8.NEO or pLV.CMV.CBG99.PURO using Fugene HD, per the manufacturer’s protocol. After 24 h, cells were lysed for 10 min with 0.4 ml of Promega’s Passive lysis buffer. Cell debris was pelleted by centrifugation at 13,000 rpm for 10 min. The level of expression of each luciferase was evaluated in triplicate, and then each luciferase was diluted to a similar level of activity in lysis buffer. Subsequently, 30 µl of each lysate were plated in linear dilutions and in different proportions and imaged after addition of 30 µl of luciferase assay buffer (Promega, Madison, WI, USA) in a 96 black-walled plate with a clear bottom. Images were taken using a set of 20 nm filter steps from 520 nm to 680 nm and without a filter:acquisition time was 2 sec and f/stop 4 at 25°C. Two independent sets of transfections and images were used for calculations. Green and red signals were calculated from unmixed images. Data were plotted using GraphPad Prism 5.

### Spectral unmixing of emission wavelengths in living cells

Stably expressing red and green HepG2 cells were trypsinized and resuspended in PBS in the 96 black-walled plates described above. Images were acquired at 37°C 5 min after addition of 1 mM D-luciferin (Synchem OHG, Felsberg, Germany) for 5 sec. Three independent experiments were carried out using the same selected cell lines.

### *In vivo* imaging in mice

HEK293 cells were plated at 2×10^5^ cells per well in a six well plate. After 24 h, 1 µg of either pLV.CMVPpy RE8.NEO or pLV.CMVCBG99.PURO was transfected as described. After 24 h, the cells were trypsinized, pelleted and resuspended in PBS at 10^5^ cells/100 µl. For injection in liver and in the dorsolateral prostate cells were resuspended in PBS at 10^5^ cells/10 µl. Aliquots were used for testing *in vitro* and *in vivo* imaging in mice.

Athymic mice (BALB/c *nu/nu*, 4–6 weeks old) mice and male Balb/c mice (8–10 weeks old) were acquired from Charles River (Charles River, L'Arbresle, France), housed in individually ventilated cages while food and water were provided *ad libitum*. Mice were anesthetized by isofluorane, while injected subcutaneously with cells. For injection of cells in the prostate and liver mice were anesthetized by Ketamine/Xylazine 100 mg/kg and 10 mg/kg body weight. Cells were implanted in the prostate or in the median liver lobe and then skin was sutured. Images were acquired 10 min after i.p. injection of D-Luciferin (150 mg/kg) using 30 sec exposure with or without filters. Four independent sets of transfections were performed and cells injected in animals as described in Table S1.

## Supporting Information

Table S1 The table describes the number of animals used for in vivo experiments regarding the injection of cells into different organs. Red, green and a mixture of red and green emitting cells could be inoculated under the skin of every mouse. For experiments carried out in the liver or in the prostate, two mice were injected in either organ with red or green emitting cells for generating reference spectra for these organs. Then mixture of red and green emitting cells were injected both in the liver and in the prostate for the evaluation of the spectral resolution of the signals.
(TIF)

## References

[pone.0019277-Kaijzel1] Kaijzel EL, van der Pluijm G, Löwik CW (2007). Whole-body optical imaging in animal models to assess cancer development and progression.. Clin Cancer Res.

[pone.0019277-Contag1] Contag CH, Bachmann MH (2002). Advances in in vivo bioluminescence imaging of gene expression.. Annu Rev Biomed ENG.

[pone.0019277-Tannous1] Tannous BA, Kim DE, Fernandez JL, Weissleder R, Breakefield XO (2005). Codon-optimized Gaussia luciferase cDNA for mammalian gene expression in culture and in vivo.. Mol Ther.

[pone.0019277-Contag2] Contag CH, Contag PR, Mullins JI, Spilman SD, Stevenson DK (1995). Photonic detection of bacterial pathogens in living hosts.. Mol Microbiol.

[pone.0019277-Close1] Close DM, Patterson SS, Ripp S, Baek SJ, Sanseverino J (2010). Autonomous bioluminescent expression of the bacterial luciferase gene cassette (lux) in a mammalian cell line.. PLoS One.

[pone.0019277-Cheong1] Cheong WF, Prahl SA, Welch AJ (1990). A review of the optical properties of biological tissues.. IEEE J Quantum Electron.

[pone.0019277-Loening1] Loening AM, Wu AM, Gambhir SS (2007). Red-shifted Renilla reniformis luciferase variants for imaging in living subjects. Nat.. Methods.

[pone.0019277-Welsh1] Welsh JP, Patel KG, Manthiram K, Swartz JR (2009). Multiply mutated Gaussia luciferases provide prolonged and intense bioluminescence.. Biochem Biophys Res Commun.

[pone.0019277-Maguire1] Maguire CA, Deliolanis NC, Pike L, Niers JM, Tjon-Kon-Fat LA (2009). Gaussia luciferase variant for high-throughput functional screening applications.. Anal Chem.

[pone.0019277-Li1] Li X, Nakajima Y, Niwa K, Viviani VR, Ohmiya Y (2010). Enhanced red-emitting railroad worm luciferase for bioassays and bioimaging.. Protein Sci.

[pone.0019277-Almond1] Almond B, Hawkins E, Stecha P, Garvin D, Paguio A (2003). Promega Note.

[pone.0019277-Branchini1] Branchini BR, Ablamsky DM, Murtiashaw MH, Uzasci L, Fraga H (2007). Thermostable red and green light-producing firefly luciferase mutants for bioluminescent reporter applications.. Anal Biochem.

[pone.0019277-Branchini2] Branchini BR, Ablamsky DM, Davis AL, Southworth TL, Butler B (2010). Red-emitting luciferases for bioluminescence reporter and imaging applications. Anal.. Biochem.

[pone.0019277-Mezzanotte1] Mezzanotte L, Fazzina R, Michelini E, Tonelli R, Pession A (2010). In vivo bioluminescence imaging of murine xenograft cancer models with a red-shifted thermostable luciferase.. Mol Imaging Biol.

[pone.0019277-Caysa1] Caysa H, Jacob R, Müther N, Branchini B, Messerle M (2009). A redshifted codon-optimized firefly luciferase is a sensitive reporter for bioluminescence imaging.. Photochem Photobiol Sci.

[pone.0019277-Gammon1] Gammon ST, Leevy WM, Gross S, Gokel GW, Piwnica-Worms D (2006). Spectral unmixing of multicolored bioluminescence emitted from heterogeneous biological sources.. Anal Chem.

[pone.0019277-Michelini1] Michelini E, Cevenini L, Mezzanotte L, Ablamsky D, Southworth T (2008). Spectral-resolved gene technology for multiplexed bioluminescence and high-content screening.. Anal Chem.

[pone.0019277-Miyawaki1] Miyawaki A (2007). Bringing bioluminescence into the picture.. Nature Methods.

[pone.0019277-Miloud1] Miloud T, Henrich C, Hämmerling GJ (2007). Quantitative comparison of click beetle and firefly luciferases for in vivo bioluminescence imaging.. J Biomed Opt.

[pone.0019277-Roda1] Roda A, Pasini P, Mirasoli M, Michelini E, Guardigli M (2004). Biothecnological applications of bioluminescence and chemiluminescence.. Trends Biotechnol.

[pone.0019277-Roda2] Roda A, Guardigli M, Michelini E, Mirasoli M (2009). Bioluminescence in analytical chemistry and in vivo imaging.. TrAC Trends in Anal Chem.

[pone.0019277-Kwon1] Kwon H, Enomoto T, Shimogawara M, Yasuda K, Nakajima Y (2010). Bioluminescence imaging of dual gene expression at the single-cell level.. Biotechniques.

[pone.0019277-Noguchi1] Noguchi T, Michihata T, Nakamura W, Takumi T, Shimizu R (2010). Dual-color luciferase mouse directly demonstrates coupled expression of two clock genes.. Biochemistry.

[pone.0019277-Zhao1] Zhao H, Doyle TC, Coquoz O, Kalish F, Rice BW (2005). Emission spectra of bioluminescent reporters and interaction with mammalian tissue determine the sensitivity of detection in vivo.. J Biomed Opt.

[pone.0019277-Wenner1] Wenner M (2009). The most transparent research.. Nat.

[pone.0019277-Sumida1] Sumida M (2007). See-through frog offers inside information.. Nature.

[pone.0019277-Xu1] Xu H, Rice BW (2009). In-vivo fluorescence imaging with a multivariate curve resolution spectral unmixing technique.. J Biomed Opt.

[pone.0019277-Hida1] Hida N, Awais M, Takeuchi M, Ueno N, Tashiro M (2009). High-sensitivity real-time imaging of dual protein-protein interactions in living subjects using multicolor luciferases.. Plos One.

[pone.0019277-Carlotti1] Carlotti F, Bazuine M, Kekarainen T, Seppen J, Pognonec P (2004). Lentiviral vectors efficiently transduce quiescent mature 3T3-L1 adipocytes.. Mol Ther.

