# Supplementary figures and images for: Sensitive Dual Color In Vivo Bioluminescence Imaging Using a New Red Codon Optimized Firefly Luciferase and a Green Click Beetle Luciferase
Source: PLoS One. 2011 Apr 22;6(4):e19277. doi: 10.1371/journal.pone.0019277 (PMC3081340; doi:10.1371/journal.pone.0019277)

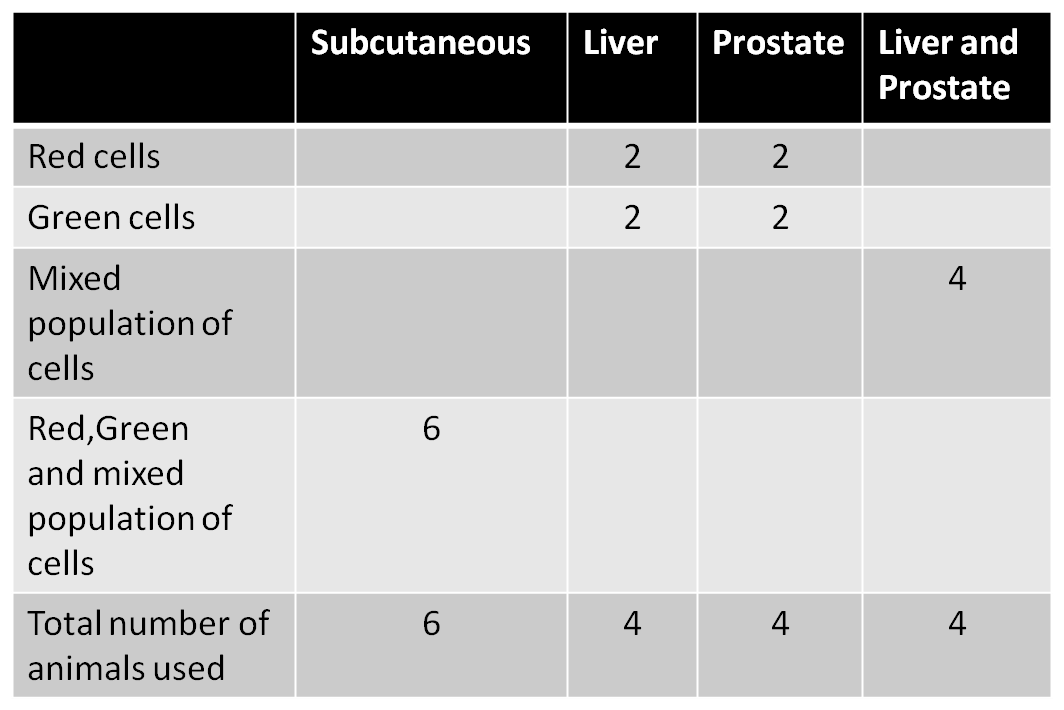

Supplement: Table S1 — The table describes the number of animals used for in vivo experiments regarding the injection of cells into different organs. Red, green and a mixture of red and green emitting cells could be inoculated under the skin of every mouse. For experiments carried out in the liver or in the prostate, two mice were injected in either organ with red or green emitting cells for generating reference spectra for these organs. Then mixture of red and green emitting cells were injected both in the liver and in the prostate for the evaluation of the spectral resolution of the signals. (TIF) [file pone.0019277.s001.tif]
